# Molecular characteristics of *Brucella melitensis* isolates from humans in Qinghai Province, China

**DOI:** 10.1186/s40249-021-00829-0

**Published:** 2021-03-26

**Authors:** Zhi-Jun Zhao, Ji-Quan Li, Li Ma, Hong-Mei Xue, Xu-Xin Yang, Yuan-Bo Zhao, Yu-Min Qin, Xiao-Wen Yang, Dong-Ri Piao, Hong-Yan Zhao, Guo-Zhong Tian, Qiang Li, Jian-Ling Wang, Guang Tian, Hai Jiang, Li-Qing Xu

**Affiliations:** 1 Qinghai Institute for Endemic Disease Prevention and Control, The department of brucellosis prevention and control, Xining, 810021 Qinghai China; 2 State Key Laboratory for Infectious Disease Prevention and Control, Collaborative Innovation Center for Diagnosis and Treatment of Infectious Diseases, National Institute for Communicable Disease Control and Prevention, Chinese Center for Disease Control and Prevention, Beijing, China; 3 Key Laboratory of Plague Prevention and Research, Qinghai Institute for Endemic Disease Prevention and Control, National Health Commission (2019PT310004) and Key Laboratory for Plague Prevention and Control of Qinghai Province, Xining, 810021 Qinghai China

**Keywords:** *Brucella melitensis*, Multiple-locus variable-number tandem repeats analysis, Whole-genome sequencing, Single-nucleotide polymorphism

## Abstract

**Background:**

The prevalence of human brucellosis in Qinghai Province of China has been increasing rapidly, with confirmed cases distributed across 31 counties. However, the epidemiology of brucellosis transmission has not been fully elucidated. To characterize the infecting strains isolated from humans, multiple-locus variable-number tandem repeats analysis (MLVA) and whole-genome single-nucleotide polymorphism (SNP)-based approaches were employed.

**Methods:**

Strains were isolated from two males blood cultures that were confirmed *Brucella melitensis* positive following biotyping and MLVA. Genomic DNA was extracted from these two strains, and whole-genome sequencing was performed. Next, SNP-based phylogenetic analysis was performed to compare the two strains to 94 *B. melitensis* strains (complete genome and draft genome) retrieved from online databases.

**Results:**

The two *Brucella* isolates were identified as *B. melitensis* biovar 3 (QH2019001 and QH2019005) following conventional biotyping and were found to have differences in their variable number tandem repeats (VNTRs) using MLVA-16. Phylogenetic examination assigned the 96 strains to five genotype groups, with QH2019001 and QH2019005 assigned to the same group, but different subgroups. Moreover, the QH2019005 strain was assigned to a new subgenotype, IIj, within genotype II. These findings were then combined to determine the geographic origin of the two *Brucella* strains.

**Conclusions:**

Utilizing a whole-genome SNP-based approach enabled differences between the two *B. melitensis* strains to be more clearly resolved, and facilitated the elucidation of their different evolutionary histories. This approach also revealed that QH2019005 is a member of a new subgenotype (IIj) with an ancient origin in the eastern Mediterranean Sea.

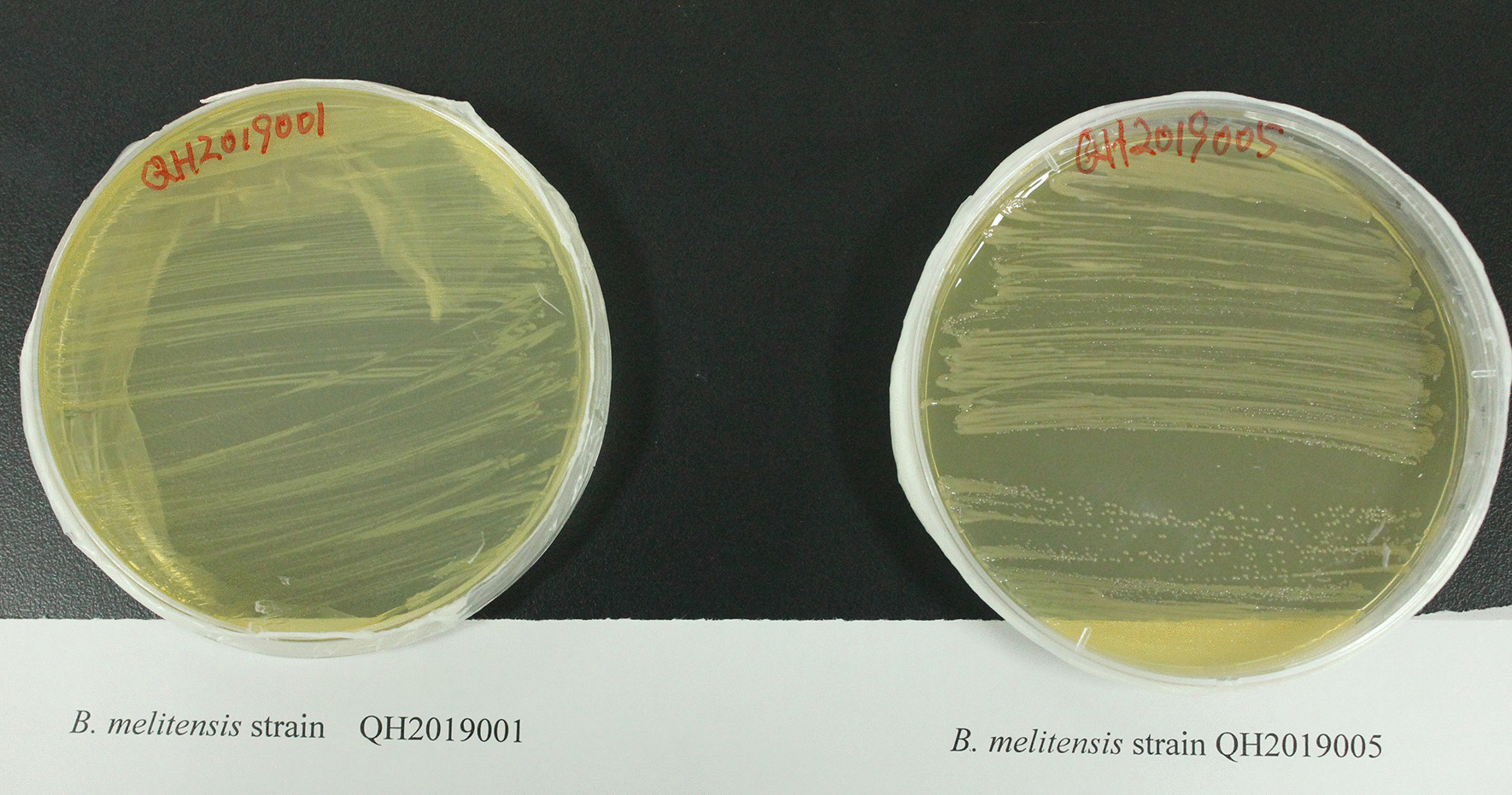

**Supplementary Information:**

The online version contains supplementary material available at 10.1186/s40249-021-00829-0.

## Background

Brucellosis, which is caused by bacteria in the *Brucella* genus, is one of the most important zoonoses worldwide and is considered a “forgotten, neglected zoonosis” by the World Health Organization [1]. This disease is endemic in regions within Africa, Asia, Latin America and other countries along the Mediterranean Sea [2, 3]. Human infections can occur due to the consumption of contaminated non-pasteurized milk or cheese, or by occupational exposure to infected animals or their carcasses, uterine secretions, or aborted fetuses [4]. While the mortality rate of brucellosis is low, the morbidity rate is much higher. Worldwide, incidence of human brucellosis varies widely, from < 0.01 to > 200 per 100 000 people in endemic disease areas [5]. In the mainland of China, the total incidence rate of human brucellosis increased from 0.92 per 100 000 people in 2004 to 4.2 per 100 000 people in 2014 [6]. Recently, in Qinghai Province, the incidence rate has increased from 0.04 per 100 000 people in 2011 to 1.96 per 100 000 people in 2018, with confirmed cases distributed across 31 counties within the entire province. Thus, brucellosis is becoming a major public health problem that impacts human physical and mental health.

*Brucella* is a gram-negative, facultative, intracellular pathogen that causes brucellosis in both humans and animals. Currently, the *Brucella* genus contains 12 accepted species, with four of those, namely, *B. melitensis*, *B. abortus*, *B. suis and B. canis* are considered zoonotic as they regularly cause humans infection [7]. While various species differ in host preference, virulence and/or zoonotic potential, most *Brucella* maintain a 97–99% genomic sequence identity [8, 9].

In humans, *B. melitensis* is the most virulent species and has been the predominant species associated with human brucellosis in China from 1953 to 2013 [10]. To ensure accurate epidemiological surveillance and to distinguish infected and vaccinated individuals, species identification and subtyping is essential [11]. Multiple loci variable-number tandem repeat analysis (MLVA) can provide the epidemiological relatedness among *Brucella* strains [12]. In a study examining *Brucella* in the Qinghai-Tibet Plateau region, none of the genotypes matched any of the sequences in the *Brucella* MLVA database (2012), possibly due to these strains having unique geographical characteristics or due to *B. melitensis* being the predominant species in that area [13]. Moreover, *Brucella* studies have also utilized whole-genome sequencing (WGS), which provides excellent genetic resolution, to resolve differences in closely related species [14]. In this study, we performed an MLVA typing of a collection of strains from the Qinghai province, 2018–2019. We selected two strains showing very distinct MLVA profiles for analysis using whole-genome single nucleotide polymorphism (SNP)-based approaches to elucidate the characteristics and homologies for epidemiological purposes. These findings will provide further insight into *Brucella* epidemiology and enable improved control and prevention of brucellosis in Qinghai Province.

## Methods

### Bacterial strains

Positive blood cultures were examined for brucellosis at the Qinghai Institute for Endemic Disease Prevention and Control in Qinghai Province, China, and deemed *Brucella*-positive between 2018 and 2019. The strains were identified as *B. melitensis* based on morphology and conventional identification methods according to standard biotyping procedures, including CO_2_ requirement, inhibition of growth by basic fuchsin and thionin, agglutination with monospecific antisera (A, M) and phage typing (Bk_2_, Tb).

Total genomic DNA was extracted from the two *B. melitensis* strains, QH2019001 and QH2019005, using a Wizard Genomic DNA Purification Kit (Promega, city, USA) according to the manufacturer’s instructions. The obtained DNA was then assessed via agarose gel electrophoresis and quantified using a Qubit 2.0 Fluorometer (Thermo Scientific, city, USA).

### Brucella MLVA-16 genotyping scheme

MLVA-16 was performed as previously described [14]. All data were analyzed using BioNumerics (version 5.1; Applied Maths, Sint-Martens-Latem, Belgium). Clustering analysis was based on the categorical coefficient and the unweighted pair group method using the arithmetic averages (UPGMA) method [15].

### Whole-genome sequencing and single-nucleotide polymorphism analyses

Sequencing libraries were generated using a NEBNext® Ultra™ DNA Library Prep Kit for Illumina (NEB, city, USA) following the manufacturer’s recommendations. WGS for the QH2019001 and QH2019005 strains was performed by Novogene Bioinformatics Technology Co., Ltd. (Beijing, China) using an Illumina NovaSeq 6000 (PE 150 bp). The raw data generated using the Illumina sequencing platform were assembled using SOAPdenovo (version 2.04). The obtained genomic sequences were available in GenBank under accession numbers of GCA 016411965.1 and GCA 016806105.1.

Genomic alignments between a sample genome and reference genome (or among more than two sample genomes) were performed by Novogene Bioinformatics Technology Co., Ltd. (Beijing, China) using the MUMmer [16] and LASTZ [17, 18] tools. Additionally, 94 *B. melitensis* genomes (Additional file 1: Table S1) were retrieved from GenBank and used for comparison and preliminary phylogenetic analyses. A phylogenetic tree was constructed using TreeBeST and PHYML (maximum-likelihood based, with 1000 bootstrap replicates utilized) [19, 20].

## Results

### Identification of *Brucella* strains

The *Brucella* strains QH2019001 and QH2019001 were identified as *B. melitensis* biovar 3 using a conventional biotyping method (Table 1).

**Table 1:** Biotyping identification results for the two *Brucella melitensis* strain

Strain IDs	CO_2_ Requirement	Sensitivity to dyes		Serum agglutination		RTD	
		Fuchin 1:25 000	Thionin1:50 000	A	M	BK2	Tb
QH2019001	-	+	+	+	+	+	+
QH2019005	-	+	+	+	+	+	+

*RTD* Routine test dilution, *BK2* Berkeley, *Tb* Tbilisi

### MLVA-16 genotyping results

**Fig. 1:**
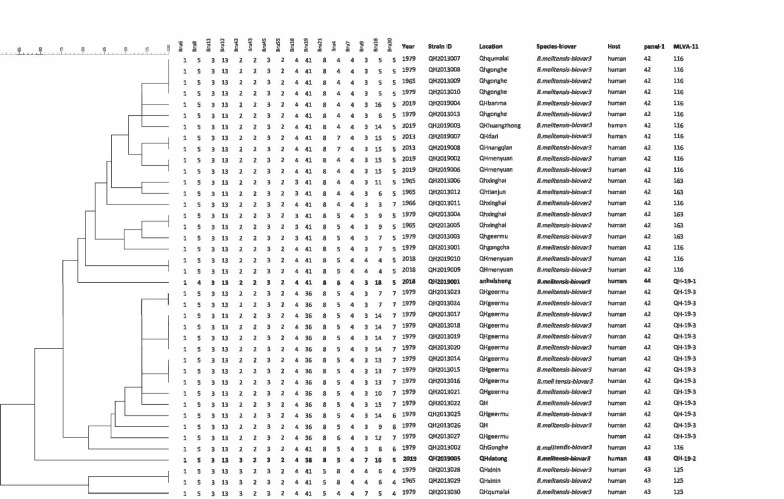
Dendrogram based on MLVA-16 genotyping

The MLVA-16 assay was used to determine the genotypes for 40 *B. melitensis* strains from human patients in Qinghai province, in 2018–2019. Two strains, QH2019001 (1–4-3–13-2–2-3–2-4–40-8–6-4–3-18–5) and QH2019005 (1–5-3–13-120 3–2-3–2-4–38-8–5-4–7-16–5) stand alone in the clustering analysis shown in Fig. 1, and define new MLVA-11 genotypes. For the two *B. melitensis* strains, variable number tandem repeat (VNTR) differences were present in bruce08 and bruce42 (panel 1) and bruce19, bruce04, bruce09 and bruce16 (panels 2A and 2B). When combining these findings with epidemiological data, *B. melitensis* strain QH2019001 appears to have been imported from Anhui Province, while the QH2019005 strain appears to be native to Qinghai Province (Fig. 1).

### Genomic characteristic and phylogenetic analysis based on whole-genome SNPs

**Fig. 2:**
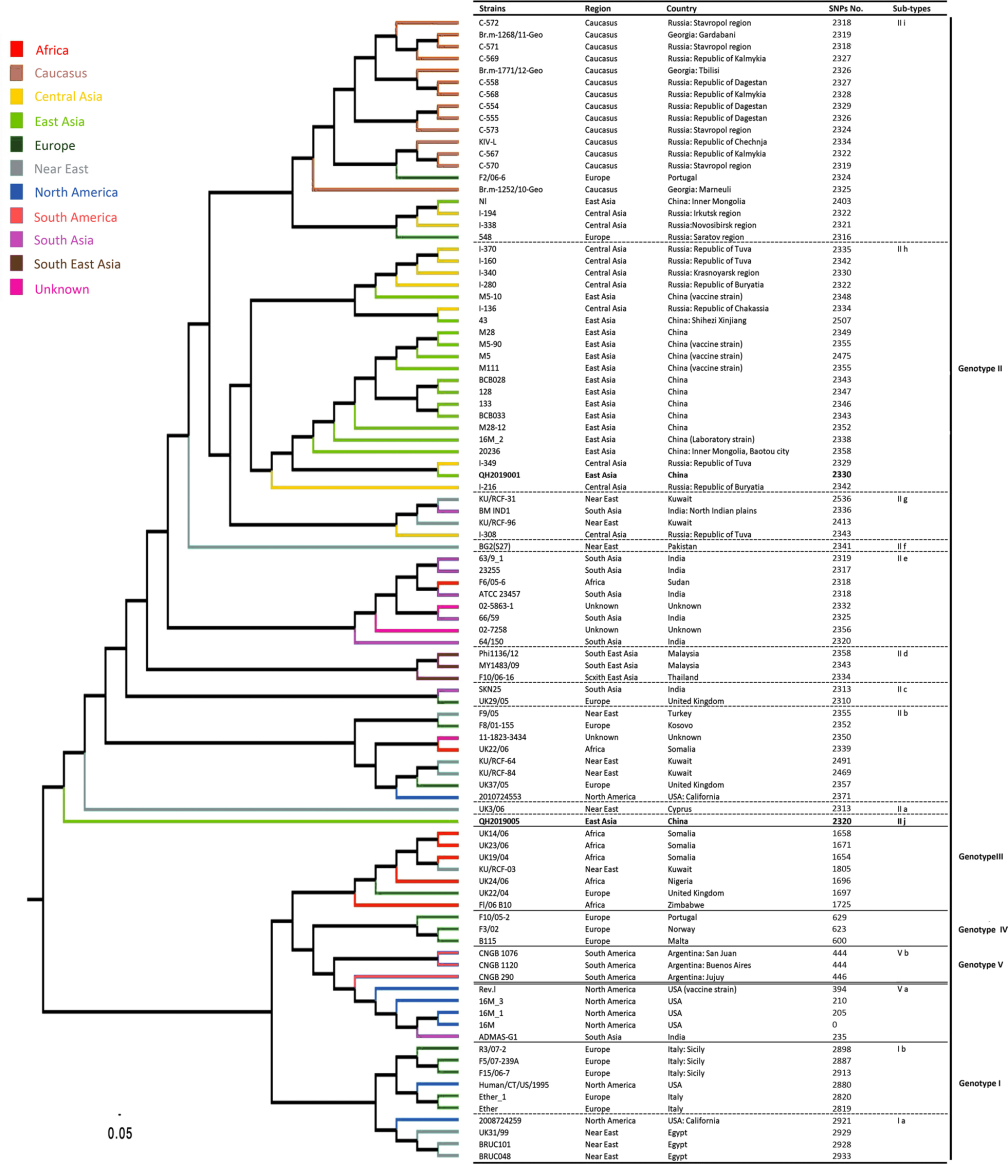
Phylogenetic tree showing the evolutionary relationships between 96 *Brucella melitensis* strains. Terminal branch colors correspond to the region where a given strain was isolated

The total contig sequence lengths were 3 291 786 bp for QH2019001 and 3 289 996 bp for QH2019005, with both having a GC content of 57.2%. To gain insight into the geographical distributions of the 96 examined *B. melitensis* strains (including standard strains and *B. melitensis* strains around the world), a phylogenetic analysis was performed using the whole-genome SNPs. The strains were divided into five major genotypes as follows: Genotype I corresponds to the West Mediterranean clade, Genotype II to the East Mediterranean clade, and genotypes III to V belong to the Americas group. Genotype III was recently designated as the African clade (strain 65/112) by Foster et al. [21] (Fig. 2). The 16M_2 strain was isolated from an infected mouse that was reported to have accumulated mutations during the infection. The position in Fig. 2 shows clearly that it is not derived from 16 M. The QH2019001 and QH2019005 strains were both associated with the genotype II group. Furthermore, several clades and subclades were isolated within the lineages and were associated with geographic attributes. The QH2019001 strain was assigned to subgenotype IIh, while the QH2019005 strain was assigned to subgenotype IIj (a new subgroup).

## Discussion

MLVA genotyping provides valuable insight when examining an epidemiological linkage or trace-back investigation during a brucellosis outbreak [22–24]. Based on the MLVA-11, the QH2019001 and QH2019005 strains formed unique genotypes and represented as single independent strain, which suggests that these two strains were not related epidemiologically. Thus the exact origins of the two strains could not be determined by MLVA. However, WGS-based analysis has been shown to distinguish very closely related *B. melitensis* strains and can discriminate intraspecies relationships [25, 26]. In this study, whole-genome SNP analysis showed that the QH2019001 and QH2019005 strains were both assigned to genotype II, but to different subgroups. Furthermore, *B. melitensis* strain QH2019005 was assigned to genotype IIj (a new subtype), which is closely associated with subgenotype IIa that contained only a single strain, UK3/06. The QH2019001 strain was assigned to subgenotype IIh. The differences between the QH2019001 and QH2019005 strains suggest that the nucleotide variation may be attributed to changes in geographic distribution. It may have been driven by the relatively isolated and more unique environment of the Qinghai-Tibet Plateau, with its extremely high altitudes. Due to the environment, it is difficult for lowland livestock breeds or wild animals to survive; hence, livestock exchange between Qinghai Province and other regions would be limited [13]. Further investigation of the movement of sheep should be performed to identify the origins of these strains.

The QH2019005 strain was the most similar to the UK3/06 strain that was isolated from the Near East, thus suggesting that this strain and its new subgenotype have ancient origins. The QH2019001 strain was the most similar to the I-349 strain that was isolated from Central Asia (Russia: Republic of Tuva) [27], thus suggesting that this strain shares a common origin. Furthermore, active trade between Russia and China could have promoted the transmission of *B. melitensis* strain QH2019001 to other regions (Anhui Province) in China.

In summary, we observed that strains in this region exhibited unique characteristics of origin and evolution. Further efforts, examination of more strains, WGS, and collection of epidemiological data from the neighboring provinces are needed to accurately outline the pattern of transmission of brucellosis in Qinghai, China.

## Conclusion

In the present study, the molecular characteristics of two human *Brucella* strains isolated from Qinghai Province were examined to gain a further understanding of the epidemiology of brucellosis. The two strains were found to have different origins and evolutionary histories, with the native strain, QH2019005, assigned to a new subgenotype with an ancient origin in the eastern Mediterranean region. Additionally, this study further highlighted that utilizing a whole-genome SNP-based approach can enable intraspecies relationships between *B. melitensis* to be more fully examined.

## Supplementary Information

**Additional file 1: Table S1.** *B. melitensis* genome information.

## Data Availability

All the relevant data have been provided within the manuscript or as a supporting file.

## Abbreviations

WGS: Whole-genome sequencing
MLVA: Multiple loci variable-number tandem repeat analysis
SNP: Single nucleotide polymorphism
SAT: Serum agglutination tests
VNTR: Variable number tandem repeat
